# Tumor cells seeding in biopsy tracts after open biopsy in three patients with chondrosarcoma—Case reports

**DOI:** 10.1007/s00508-025-02677-6

**Published:** 2025-12-23

**Authors:** Teodora Todorova, Maria Anna Smolle, Magdalena Maria Gilg, Bernadette Liegl-Atzwanger, Jasminka Igrec, Christian Viertler, Andreas Leithner

**Affiliations:** 1 Department of Orthopaedics and Trauma, Medical University of Graz, Auenbruggerplatz 5, 8036 Graz, Austria; 2 University Clinic for Orthopedic Surgery, Skopje, North Macedonia; 3 Department of Orthopedics, Centre of Rheumatology, Oberammergau, Germany; 4 Diagnostic and Research Institute of Pathology, Medical University of Graz, Graz, Austria; 5 Division of General Radiology, Department of Radiology, Medical University of Graz, Graz, Austria

**Keywords:** Open biopsy, Tumor cell seeding, Biopsy tract, Chondrosarcoma, Local recurrence

## Abstract

**Purpose:**

The biopsy represents a fundamental step towards diagnosing bone and soft tissue tumors. In the case of open biopsies it is recommended to resect the biopsy tract at wide resection of bone and soft tissue sarcomas (STS); however, nowadays this topic is subject to ongoing debate as a worldwide shift from open to core-needle biopsies may lead to a decreased risk of local recurrence (LR) in the biopsy tract.

**Methods:**

We present three patients (male, 69 years; male, 63 years; female 76 years) with chondrosarcomas treated at the Department of Orthopaedics and Trauma, Medical University of Graz: two with sarcomas involving the distal femur and one in the proximal femur. Staging showed no evidence of distant metastases (DM). Wide resection and reconstruction with modular tumor endoprostheses were performed in all three patients with the biopsy tract included in the resection specimen.

**Results:**

In all three patients, histological analysis of the resected specimen revealed viable tumor cell nests in the biopsy tract after an open biopsy technique.

**Conclusion:**

The three presented cases demonstrate that tumor cell seeding occurs in biopsy tracts after an open biopsy in chondrosarcoma cases. Whether the risk of developing LR from these tumor cells differs between open and minimally invasive biopsy techniques or is affected by the use of neoadjuvant chemotherapy and/or radiotherapy for other entities, requires larger cohorts and longer periods of follow-up.

## Introduction

The biopsy is a key step for accurate diagnosis and identification of the histological subtype of bone and soft tissue tumors. Adequate tissue samples are therefore required, ideally obtained without excessive manipulation of the lesion [1, 2]. It is subject to ongoing debate whether removal of the biopsy tract during the definitive resection of a malignant tumor is necessary, especially after minimally invasive biopsy techniques [3, 4]. In general, one of the two types of biopsy techniques is used to obtain tissue sample: closed (percutaneous) biopsies in which the tissue sample is obtained via a small incision and needles, and open biopsy (incisional or excisional) in which tissue sample is obtained through a surgical approach [9]. In 1987 Cannon and Dyson [5] reported a lower LR rate in cases where an open biopsy technique was used and the biopsy tract was resected, compared to cases where the biopsy tract remained untouched. The perception of possible contamination of the biopsy tract with tumor cells among the community of orthopedic oncologists seemed to be reinforced with this study.

We report on three patients with chondrosarcomas, in whom the histological analysis of the specimen revealed viable tumor cell nests in the biopsy tract following an open biopsy.

## Patients, material and methods

In this case study, three patients (male 69 years; chondrosarcoma of the right distal femur; male 63 years; chondrosarcoma of the left distal femur; female 76 years, chondrosarcoma of the left proximal femur) treated at a single sarcoma center were retrospectively included. Preoperative staging revealed no evidence of distant metastases. In all patients, wide resection and reconstruction with a modular tumor endoprosthesis were performed. Histological analysis of the resected specimen revealed viable tumor cell nests in the biopsy tract after an open biopsy technique in all three patients. This study was approved by the local institutional review board (IRB-number: 1152/2024).

## Results

### Patient 1

**Fig. 1:**
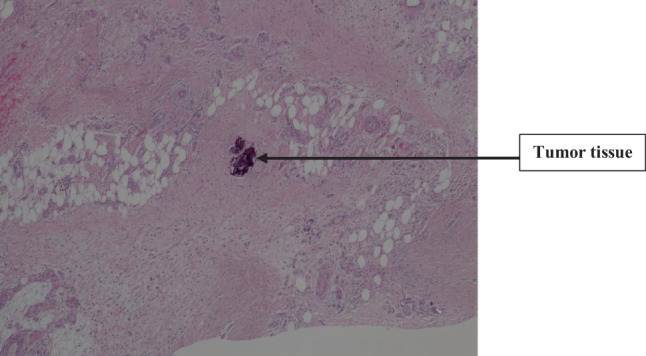
Microscopic appearance of fragments of cartilaginous proliferation in the biopsy tract, H&E stain, 40x magnification

**Fig. 2:**
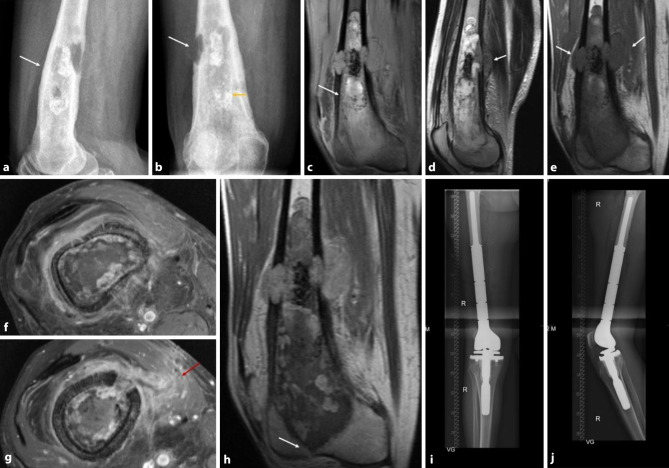
(Case 1). Anteroposterior (AP) and lateral radiographs of the distal femur showing an osteoblastic-osteolytic lesion (**a**, white arrows) with poorly defined margins and subtle periosteal reaction at the medial aspect of the distal femur. The lesion demonstrates a broad transition zone with a cartilaginous matrix (yellow arrow), cortical destruction and thinning (**b**). Coronal and sagittal T2-weighted MRI with and without fat saturation showing a heterogeneous osteodestructive lesion with typical nodular enhancement and significant mucoid component, surrounding bone marrow oedema (**c**) in the distal femur with cortical disruption (**d**). (**e**) Extraosseous tumor component along the bone circumference with low signal intensity. Postcontrast axial T1-weighted fat-saturated MRI sequences on two consecutive slices showing the extent of the lesion with circumferential cortical infiltration, heterogeneous contrast enhancement of the extraosseous tumor component in the prefemoral fat and biopsy tract on the medial side of the upper leg (red arrow) (**f**, **g**). The distal extension of the tumor without the signs of joint infiltration (**h**). Postoperative radiographs in AP and lateral after wide resection with built-in modular tumor endoprosthesis (**i**, **j**)

A 69-year-old male patient presented to an external hospital with right knee pain, which had started 1.5 years before the examination, without a history of previous trauma. The diagnostic work-up included radiographs, a computed tomography (CT) scan, magnetic resonance imaging (MRI) of the knee, and a Tc99m bone scintigraphy scan. The biopsy revealed the presence of an atypical cartilaginous tumor (ACT), formerly known as chondrosarcoma G1. The patient was subsequently referred to our department. A follow-up MRI of the right knee revealed cortical disruption and a soft tissue mass highly suggestive of high-grade chondrosarcoma. Staging showed no evidence of DM. Given the discrepancy between histology and imaging, the multidisciplinary tumor board considered the possibility of a sampling error. Thus, 3 weeks after the biopsy, wide resection and reconstruction of the distal femur with a modular tumor endoprosthesis were performed (Fig. 2). The biopsy tract was included in the definitive resection specimen. Histological analysis of the surgical specimen confirmed the presence of dedifferentiated chondrosarcoma G3, while in the area of the biopsy tract, nests of viable chondrosarcoma cells were detected (Fig. 1). Resection margins were tumor-free. After the surgery, the patient returned to his home country and was lost to follow-up.

### Patient 2

**Fig. 3:**
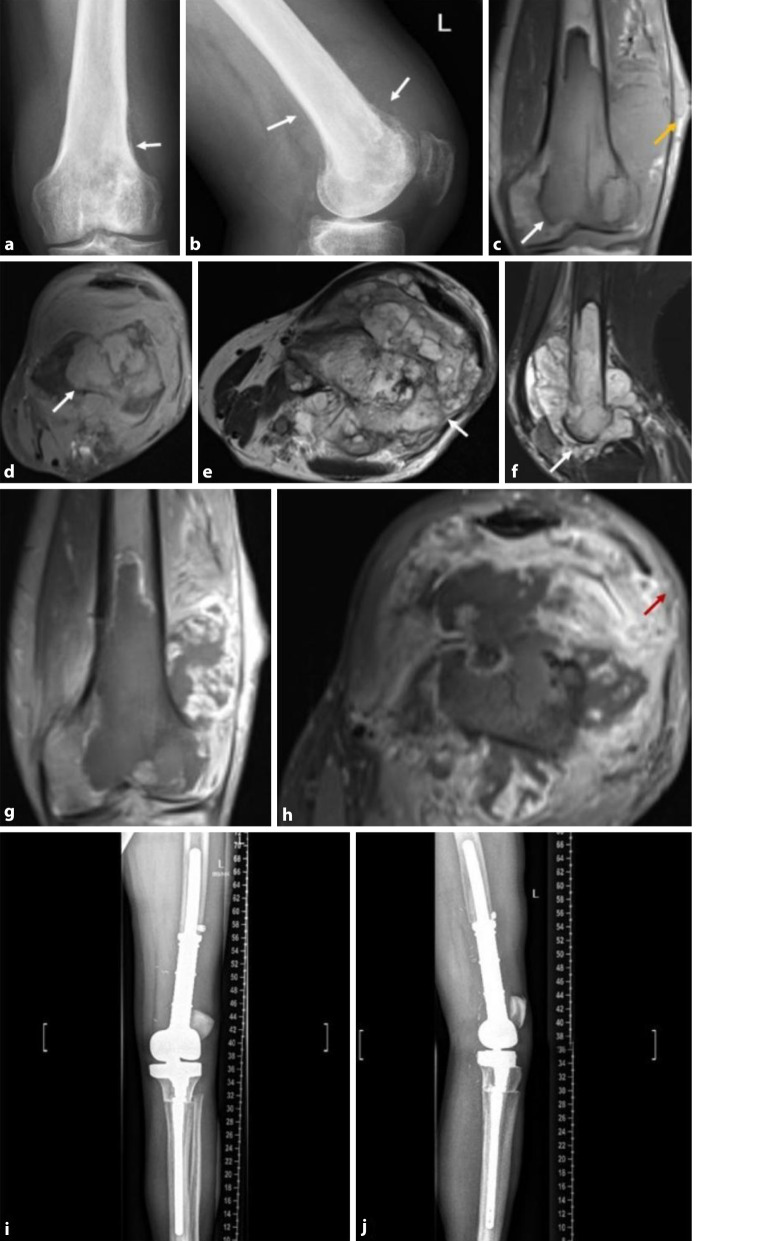
Case 2. **a**, **b** Anteroposterior (AP) and lateral radiographs of the distal femur showing a heterogeneous bone structure with aggressive periosteal reaction along the distal medial metadiaphysis (**a**, arrow) and pressure erosion. On MRI, the lesion shows hyperintense appearance on T1-weighted images (**c**, **d**) and heterogeneous hyperintense signal on T2-weighted images with circumferential cortical destruction (**e**) and joint infiltration (**f**). After intravenous contrast administration (**g**, **h**) typical nodular enhancement of cartilaginous tumor tissue is shown with biopsy tract (**h**, red arrow) on the medial side. **i**, **j** Postoperative radiographs in AP (**i**) and lateral view (**j**) after wide resection with modular tumor knee endoprosthesis

A 63-year-old male patient presented with pain and persisting swelling around the left knee joint, which had started 3 months before. He had no history of a previous trauma and X‑ray examination revealed a periosteal reaction on the lateral femoral condyle with osteoplastic and osteolytic lesions (Fig. 3). The use of MRI with gadolinium contrast medium confirmed the presence of a tumor of the distal femur with partial destruction of the cortex and extensive tumor formation in the surrounding soft tissue highly suggestive of chondrosarcoma (Fig. 3).

**Fig. 4:**
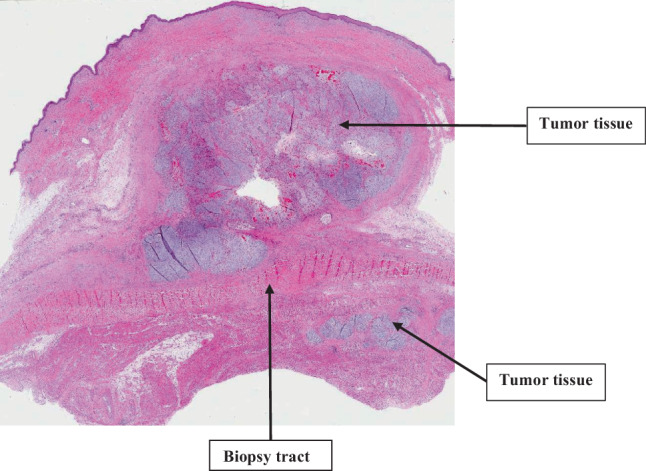
Case 2. Microscopic appearance of the resected specimen, H&E stain, 40x magnification, showing viable chondrosarcoma cells (arrow) infiltrating along the biopsy tract

Staging was negative for DM. An open biopsy showed hypercellular cartilaginous proliferation with significant nuclear atypia, confirming G3 chondrosarcoma. Wide extra-articular resection, including the biopsy tract and reconstruction using a modular tumor knee endoprosthesis with proximal tibia allograft, was performed. Histological analysis of the surgical resection specimen confirmed the diagnosis of a poorly differentiated chondrosarcoma G3 with joint invasion. Tumor cells were also found in the biopsy tract (Fig. 4).

**Fig. 5:**
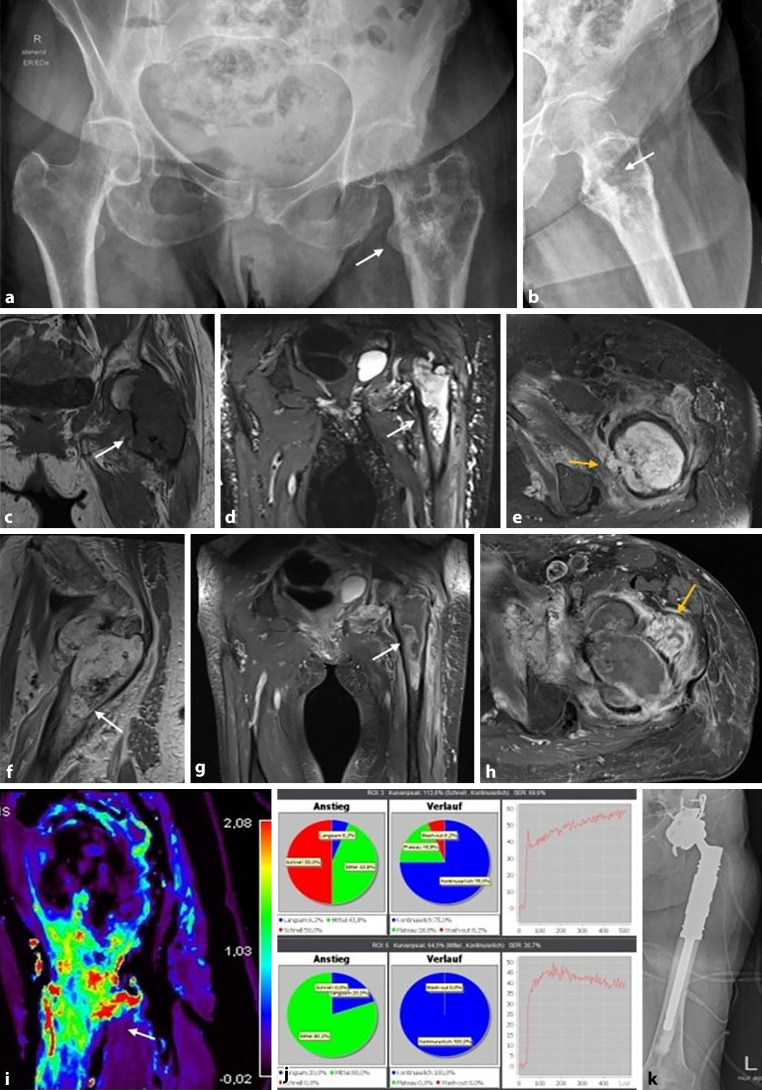
Case 3. AP radiography of the pelvis (**a**) and oblique projection of the left hip joint (**b**) show an osteolytic lesion in the trochanteric area with a cartilaginous matrix. On precontrast MRI, hypointense signal of the lesion (**c**) with cervical and diaphyseal extension of the tumor in the coronal T2-weighted image with a significant mucoid component. Axial T2-weighted image (**e**) with fat saturation shows the intraarticular extraosseous component of the tumor with surrounding bone marrow oedema on sagittal T2-weighted image (**f**). After contrast administration on coronal and axial T1-weighted images, nodular enhancement and typical lobular appearance of the tumor are shown (**g**, **h**). On dynamic contrast-enhanced MRI, diffuse patchy areas with high proliferation rate and time intensity curve Type 4 and Type 5 (**i**, **j**). Postoperative radiography of the lower extremity with tumor hip endoprosthesis (**k**)

**Fig. 6:**
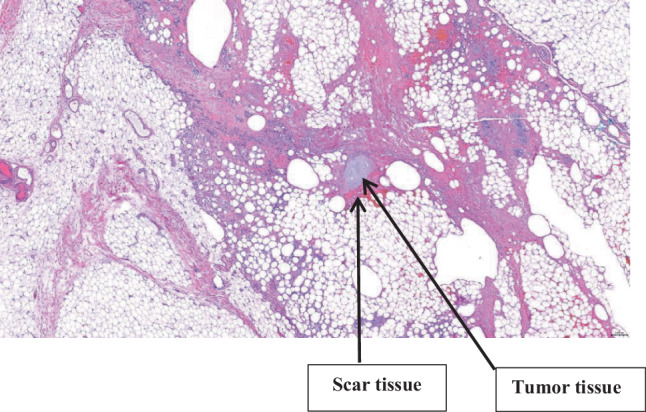
Case 3. Microscopic appearance of the resected specimen, H&E stain, 5.0x magnification showing viable chondrosarcoma cells (arrow) infiltrating along the biopsy tract

### Patient 3

A 76-year-old female patient presented with pain in the left hip responsive to pain medication. The pain had started 5 months before. Radiographic examination with MRI and X-ray was performed, revealing an osteolytic lesion of the left proximal femur with calcifications. A chondrosarcoma (at least G2) of the proximal femur was diagnosed via an open biopsy. Staging revealed no evidence of distant metastases. As there was clear radiological evidence of hip joint invasion, an extra-articular hip joint resection and proximal femoral resection were carried out (Fig. 5, 6). At the last follow-up 9 months after surgery the patient had undergone a reoperation due to recurrent dislocation of the hip but had no evidence of disease.

## Discussion

These three cases clearly demonstrate that tumor cell seeding occurs in the biopsy tract following open biopsies in patients with chondrosarcomas.

There are several potential risk factors for tumor cell contamination of biopsy tracts. One hypothesis is that multiple attempts to obtain tissue samples in open and core needle biopsies are associated with a higher risk of tumor cell seeding in the biopsy tract [1, 7]. Another hypothesis is that percutaneous as compared to open biopsy techniques are associated with a lower risk of contamination of the biopsy tract due to less tissue manipulation, resulting in less bleeding [4, 6].

Barrientos-Ruiz et al. [7] concluded that contamination of the biopsy path occurred in both open and percutaneous biopsy techniques but was more likely to occur in open biopsies, regardless of the center where the biopsy had been carried out; however, in the study open biopsies were only performed after inconclusive percutaneous biopsies, which introduces bias as there is an overall increased risk of tumor cell seeding following multiple biopsy attempts.

Mohana et al. [8] reported tumor cell contamination of the biopsy tract in 17 out of 26 osteosarcoma patients who underwent either open or core needle biopsies. This study specifically examined biopsy tract specimens obtained during definitive tumor resection and assessed them for the presence of tumor cells. The authors found that tumor grade did not significantly influence the risk of contamination, although all grade III lesions demonstrated evidence of seeding.

Ribeiro et al. [9] found biopsy tract contamination in 15 from 49 cases of malignant musculoskeletal tumors (including osteosarcomas, Ewing’s sarcomas and STS), in whom the biopsy tract was routinely examined after resection. In this study, 19 percutaneous biopsies and 30 open biopsies were investigated, with contamination occurring in 40% of open biopsies compared to 15.8% of percutaneous biopsies, highlighting the increased risk associated with open procedures; however, in both studies there was no evidence of LR after removing the biopsy tract, despite the high rates of histologically confirmed contamination during follow-up periods, suggesting proper resection of the contaminated tract may be protective against LR.

Some studies on various sarcoma histologies suggest that neoadjuvant radiotherapy may decrease tumor cell seeding in the biopsy tract. Neoadjuvant radiotherapy was not an independent risk factor in the study of Barrientos-Ruiz et al. [7]; however, for those tumors not responsive to chemotherapy and/or radiotherapy like chondrosarcoma and chordoma, one might presume that an iatrogenic spread of tumor cells in a unresected biopsy tract is more likely to lead to a clinically apparent LR.

Whether to include the biopsy tract in the definitive resection specimen is currently subject to ongoing debate, as a worldwide shift from open biopsies to trocar/core-needle biopsies may lead to a decreased risk of LR in the biopsy tract.

In conclusion, the three presented cases demonstrate that tumor cell seeding occurs in biopsy tracts after an open biopsy in chondrosarcoma cases. The clinical significance of the findings regarding LR risk cannot be definitely established from these cases alone, as the biopsy tract was resected in all three cases. Whether the risk of developing LR from these tumor cells differs between open and minimally invasive biopsy techniques or is affected by the use of neoadjuvant chemotherapy and/or radiotherapy for other entities, requires larger cohorts and longer periods of follow-up.

## Data Availability

The datasets generated and/or analyzed during the current study are not publicly available due to data protection policies but are available from the corresponding author on reasonable request.
